# Long-term follow-up after active surveillance or curative treatment: quality-of-life outcomes of men with low-risk prostate cancer

**DOI:** 10.1007/s11136-017-1507-7

**Published:** 2017-02-06

**Authors:** Lionne D. F. Venderbos, Shafak Aluwini, Monique J. Roobol, Leonard P. Bokhorst, Eric H. G. M. Oomens, Chris H. Bangma, Ida J. Korfage

**Affiliations:** 1 Department of Urology, Erasmus University Medical Center, Room Na1710, P.O. Box 2040, 3000 CA Rotterdam, The Netherlands; 2 Department of Public Health, Erasmus Medical Center, Rotterdam, The Netherlands; 3 Department of Radiology, Erasmus Medical Center, Rotterdam, The Netherlands; 4 Department of Urology, Amphia Hospital, Breda, The Netherlands

**Keywords:** Active surveillance, Quality of life, Patient reported outcome, Radical prostatectomy, Radiotherapy, Shared decision-making

## Abstract

**Purpose:**

To compare long-term (4–10 years) quality of life (QoL) of men with low-risk prostate cancer (PCa) treated by different modalities and a reference group without PCa.

**Methods:**

In this cross-sectional study, four groups were sent a one-time QoL-questionnaire; PCa patients (1) following the structured Prostate cancer Research International Active Surveillance protocol, (2) who underwent radical prostatectomy (RP) in the context of the European Randomized study of Screening for Prostate Cancer—section Rotterdam, (3) who underwent radiotherapy (RT) at an academic hospital in The Netherlands, and (4) an age-matched reference group of men without PCa. The QoL-questionnaire addressed prostate-specific health (EPIC), generic health (SF-12), and anxiety (STAI-6). Statistical significance (*p* ≤ 0.05) and clinical relevance (≥0.5 SD) of differences between groups were assessed.

**Results:**

The AS, RP, RT, and reference group response rates amounted to 74% (122/165), 66% (70/106), 66% (221/335), and 75% (205/273), respectively. At a mean of 6.6 years of follow-up, active surveillance (AS)-men reported better urinary function [*M* = 93.0 (SD = 10.6) vs. 80.0 (SD = 19.1), *p* ≤ 0.001], less urinary incontinence [*M* = 90.0 (SD = 14.6) vs. 70.1 (SD = 28.8), *p* ≤ 0.001], and better sexual function [*M* = 40.9 (SD = 24.6) vs. 14.8 (17.7), *p* ≤ 0.001, clinically relevant] than RP-men. Compared to RT, AS-men reported better sexual function [*M* = 40.9 (SD = 24.6) vs. 25.8 (SD = 25.0), *p* = 0.069]. The four groups reported similarly low anxiety levels; the number of highly anxious men (STAI ≥ 44) ranged from 8 to 13%. For all QoL domains, men on AS and men without PCa reported very similar scores.

**Conclusions:**

Prostate-specific function of AS-men was significantly better than that of RP-men. When comparing AS to RT, a borderline significant difference in sexual function was seen. Men who followed an AS strategy for a long-term period were not anxious and accepted it well, suggesting that AS may be a good treatment option for men with low-risk PCa.

## Introduction

Men diagnosed with low-risk prostate cancer (PCa) generally have three therapy options: radical prostatectomy (RP), radiotherapy (RT), or active surveillance (AS). Curative therapies, like RP and RT, are associated with side-effects, such as incontinence and impotence, while AS is aimed at deferring such side-effects by opting for an initial monitoring strategy [1]. With AS initial curative therapy is delayed, or avoided, and replaced by regular follow-up visits using prostate-specific antigen (PSA), digital rectal examination (DRE), prostate biopsy, and—potentially—magnetic resonance imaging (MRI). Choosing AS, however, requires living with untreated cancer and coping with the possibility of missing the ‘window of curability’.

AS is reported to be safe [2, 3]. In a 15-year time frame, among men that underwent AS in the Sunnybrook cohort, 2.8% developed metastases and 1.5% died of PCa; this mortality rate being consistent with that of favourable-risk patients managed with the initial curative therapy [4]. AS is now included in many guidelines as a treatment option for men diagnosed with low-risk PCa, and over the years, the number of men choosing AS has been rising [2, 3, 5–7]. The need to understand the effect of AS on well-being of men is becoming more apparent now.

In a recent systematic review, Bellardita and colleagues concluded that, so far, no major perturbations in the health-related quality of life (QoL) and psychological well-being of men on AS were seen over a follow-up period of 9–36 months [8]. However, QoL research amongst men on AS is still scarce compared to RP and RT QoL research, and current AS studies show some methodological drawbacks like the infrequent use of comparator groups, the lack of an appropriate non-cancer control group, and the use of various QoL measures which hinders comparison of QoL across treatment groups [8–10]. Furthermore, long-term patient reported outcomes are scarce. We aim to fill this gap by assessing long-term, i.e. 4–10 year, QoL of men with low-risk PCa who were either treated with AS, RP, or RT. We included an age-matched group of men without PCa as a reference group. All groups completed the same set of QoL measures. With the outcomes of this study, we would like to support patients and clinicians in weighing the advantages and disadvantages of therapies for low-risk PCa in terms of QoL, enabling an upfront informed treatment-choice which better reflects patients’ expectations and preferences relating to (side) effects of treatment [11].

## Patients and methods

### Study population

In this cross-sectional study, we compared QoL of early diagnosed, low-risk PCa patients and a reference group of men without cancer.

1. *AS*: Men who were diagnosed with low-risk PCa in the ERSPC trial (20%) or in clinical practice (80%) choose AS and participated in the Prostate cancer Research International: Active Surveillance (PRIAS) study [12]. Its inclusion criteria are: Prostate-Specific Antigen (PSA) ≤10 ng/ml, PSA-density <0.2, clinical Tstage T1c-T2, Gleason score 3 + 3 = 6, one or two biopsy cores invaded with PCa [13]. 165 participants from 11 Dutch hospitals (academic or non-academic) with ≥4 years of follow-up and still on AS were invited to participate.
2. *RP*: men who were diagnosed with low-risk PCa in the screening arm of the European Randomized study of Screening for Prostate Cancer (ERSPC), Rotterdam [14]. The 106 men invited to participate in this QoL study were diagnosed with Gleason ≤7 PCa, underwent RP, and had ≥4 years of follow-up.
3. *RT*: men who were diagnosed with low-risk PCa in the ERSPC trial (37%) or in clinical practice (63%) and underwent RT (HDR-brachytherapy as boost followed by external radiation, HDR-brachytherapy as monotherapy, or stereotactic body RT with Cyberknife) in Erasmus Medical Center, Rotterdam. 335 patients with ≥ 4 years of follow-up were invited to participate in this QoL study.
4. *Reference group without PCa*: 273 ERSPC Rotterdam screening arm participants were randomly selected from a group of 1251 who were last screened in 2012–2014. In a period of ≥15 years, participants were screened four or five times and no PCa was found. Based on date of birth, this group was age-matched to the AS, RP, and RT groups.

Men were sent an informed consent form and a one-time QoL-questionnaire in February 2015. After 1 month, one reminder was sent. This study was approved by the Institutional Review Board of the Erasmus University Medical Center (MEC number 2014-596).

### Measures included in the questionnaire

A set of validated measures was used to evaluate prostate-specific health (Expanded Prostate cancer Index Composite—EPIC), generic health (Short-Form Health-Survey—SF-12, EQ visual analogue scale—EQ-VAS), and generic anxiety (State-Trait Anxiety Inventory—STAI-6).

The EPIC [15, 16] includes 21 items on function and bother in the urinary, bowel, and sexual domains with scores ranging from 0 to 100. Higher scores reflect better functioning. Because incontinence is more related to RP and irritative/obstructive symptoms are more related to RT, we also assessed the incontinence and irritative/obstructive subscales [16]. We furthermore report distinctive items from the urinary and sexual domains because of their relevance in daily clinical practice for both patient and physician.

The SF-12 consists of 12 items with which the physical (PCS) and the mental component summary score (MCS) can be calculated, both ranging from 0 to 100, with 100 indicating best health [17]. In addition, the EQ-VAS was used, recording self-rated health on a vertical ‘thermometer’ with endpoints labelled as 100, indicating ‘best imaginable health state’ and 0, indicating ‘worst imaginable health state’ [18].

Finally, generic anxiety scores assessed through the STAI-6 range from 20 to 80; a higher score indicates more anxiety. An individual is considered highly anxious in case of a STAI-6 score ≥44 [19, 20].

### Statistical analysis

We report mean and standard deviation (SD) of patient reported outcomes per group. First, the significance of differences in terms of QoL and symptoms between all groups were tested using ANOVA. To assess which groups were statistically significantly different from each other, the ANOVA Tukey post hoc test was performed. Exploring differences further, we compared outcomes between the three treatment groups using ANCOVA (Bonferroni post hoc test), including the covariates age, PSA at diagnosis, cTstage, Gleason score, and follow-up time. *P* values (two-sided) ≤0.05 were considered statistically significant. The clinical relevance of observed differences was assessed with differences ≥0.5 SD considered clinically relevant [21]. Analyses were performed with SPSS, version 21 (IBM, Armonk, NY, USA).

## Results

**Fig. 1:**
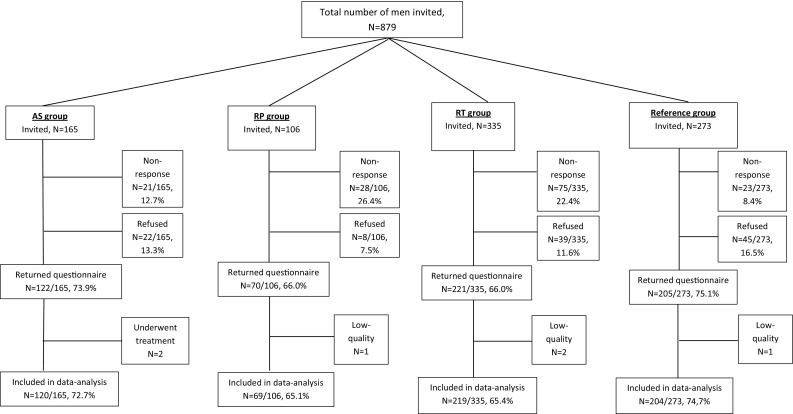
Patient cohort selection

The AS, RP, RT, and reference group response rates amounted to 74% (122/165), 66% (70/106), 66% (221/335), and 75% (205/273), respectively (Fig. 1). Patterns in the non-response/missing data did not significantly differ between participants vs. non-participants. Overall, data of four patients were excluded because of completion of ≤50% of questions and data of two men on AS were excluded, as they no longer followed the AS protocol at the time of questionnaire completion. Table 1 depicts participants’ clinical characteristics at time of diagnosis and demographic characteristics at time of questionnaire sending. Participants’ ages at time of questionnaire sending ranged from 72 to 76 years (SD = 1.6–6.4 years) and the mean follow-up time ranged from 6 to 8.6 years (SD = 1.5–3.4 years). The AS group had somewhat more favourable clinical characteristics at diagnosis than the radical treatment groups.

**Table 1:** Medical and demographic characteristics of participants at the moment of diagnosis, by treatment type

	AS	RP	RT	Reference group	*P* value*
Number of participants	120	69	219	204	
Age at treatment (mean, SD)	65.3 (6.4)	70.0 (2.6)	65.9 (6.2)		<0.001
Age at time of questionnaire (mean, SD)	71.9 (6.4)	76.0 (2.6)	74.5 (6.4)	74.5 (1.6)	<0.001
Follow-up time in years (mean, SD)	6.6 (1.6)	6.0 (1.5)	8.6 (3.4)		<0.001
PSA at diagnosis (mean, SD)	5.9 (3.1)	7.3 (6.1)	7.5 (3.1)		<0.001
Clinical stage at diagnosis (*n*, %)					
T1c	98 (81.7%)	44 (66.7%)	147 (68.1%)		0.012
T2a	22 (18.3%)	22 (33.3%)	72 (33.3%)		
Missing	0	3	3		
Gleason score at diagnosis (*n*, %)					
4	2 (1.7%)	0	0		<0.001
5	3 (2.5%)	2 (2.9%)	4 (2.1%)		
6	115 (95.8%)	46 (66.7%)	170 (89.0%)		
7	0	21 (30.4%)	17 (8.9%)		
Missing	0	0	28		
Education (*n*, %)					
Low	30 (25.4%)	21 (31.3%)	57 (26.6%)	73 (35.8%)	0.002
Intermediate	41 (34.7%)	33 (49.3%)	89 (41.6%)	82 (40.2%)	
High	44 (37.3%)	13 (19.4%)	62 (29.0%)	43 (21.1%)	
Other	3 (2.5%)	0	6 (2.8%)	6 (2.9%)	
Missing	2	2	5	0	
Working status (*n*, %)					
Employed	15 (12.5%)	2 (3.0%)	16 (7.4%)	4 (2.0%)	0.001
Retired	103 (85.8%)	63 (95.5%)	193 (89.4%)	199 (97.5%)	
Other	2 (1.7%)	1 (1.5%)	7 (3.2%)	1 (0.5%)	
Missing	0	3	3	0	
Civil status (*n*, %)					
Married/living together	105 (87.5%)	53 (79.1%)	181 (83.0%)	177 (86.8%)	0.304
Other	15 (12.5%)	14 (20.9%)	37 (17.0%)	27 (13.2%)	
Missing	0	2	1	0	

*Significance tested with ANOVA for all four groups where applicable and three groups for the clinical characteristics

### Prostate-specific health (EPIC)

#### Urinary domain

The AS (M = 93.0, SD = 10.0) and RT (M = 89.7, SD = 16.2) groups reported significantly better urinary function than the RP group (M = 80.0, SD = 19.1) [*F* (3,608) = 16.2, *p* < 0.001] (Table 2).

**Table 2:** Quality of life in the active surveillance (AS), radical prostatectomy (RP), radiotherapy (RT), and reference groups

	Score range	AS	RP	RT	Reference	All groups	AS vs. RP	AS vs. RT	AS vs. Ref.	RP vs. RT
		Mean (SD)	Mean (SD)	Mean (SD)	Mean (SD)	*P* value*	*P* value^#^	*P* value^#^	*P* value^#^	*P* value^#^
Generic health										
SF12-PCS	0–100	50.7 (6.6)	48.4 (7.7)	47.8 (8.3)	48.7 (7.7)	*0.023*	0.317	*0.011*	0.173	0.910
SF12-MCS	0–100	54.5 (9.4)	52.0 (9.2)	54.6 (8.5)	53.1 (9.6)	0.163	0.451	0.994	0.665	0.255
Respondent’s self-reported health										
EQ-VAS	0–100	81.8 (12.7)	78.6 (15.0)	78.3 (14.1)	79.5 (12.4)	0.145	0.355	0.122	0.546	1.00
Generic anxiety										
STAI-6	20–80	31.4 (8.4)	33.5 (10.5)	33.2 (8.6)	32.3 (9.4)	0.195	0.508	0.199	0.862	1.00
Highly anxious, *N* (%)	≥44	10 (8.3%)	9 (13.0%)	27 (12.3%)	24 (11.8%)	0.746	0.374	0.327	0.320	0.903
Prostate-specific health										
EPIC										
Urinary summary	0–100	91.3 (10.0)	85.6 (14.4)	89.6 (14.7)	92.1 (9.7)	*0.002*	*0.006*	0.094	0.963	0.342
Urinary function	0–100	93.0 (10.6)	80.0 (19.1)	89.7 (16.2)	93.3 (9.5)	<*0.001*	<*0.001*	0.087	0.995	<*0.001*
Urinary bother	0–100	90.0 (11.8)	89.9 (13.1)	89.4 (15.1)	91.2 (12.1)	0.889	0.992	0.977	0.999	0.920
Urinary incontinence	0–100	90.0 (14.6)	70.1 (28.8)	86.5 (20.3)	90.4 (13.9)	<*0.001*	<*0.001*	0.428	0.999	<*0.001*
Urinary irritative	0–100	92.7 (8.9)	95.5 (7.2)	92.2 (12.6)	93.7 (8.8)	*0.043*	0.475	0.509	1.00	*0.034*
Bowel summary	0–100	96.7 (6.4)	94.6 (8.0)	94.7 (7.8)	96.1 (6.9)	0.996	0.997	1.00	1.00	0.995
Bowel function	0–100	95.7 (6.0)	92.6 (9.2)	93.0 (9.1)	94.4 (8.5)	0.712	0.929	0.662	0.793	0.993
Bowel bother	0–100	97.7 (7.7)	96.6 (8.3)	96.4 (7.7)	97.8 (6.3)	0.640	0.955	0.622	0.687	0.974
Sexual summary	0–100	53.9 (20.1)	34.2 (14.9)	41.1 (20.2)	49.8 (20.1)	*0.008*	*0.008* ^‡^	0.228	0.874	0.221
Sexual function	0–100	40.9 (24.6)	14.8 (17.7)	25.8 (25.0)	35.3 (24.7)	<*0.001*	<*0.001* ^‡^	0.069	0.716	*0.045*
Sexual bother	0–100	83.2 (23.4)	77.8 (30.2)	75.8 (26.7)	83.7 (23.8)	0.873	0.995	0.998	0.955	0.978

*SD* standard deviation

*Significance tested with ANOVA for all four groups

^#^ANOVA Tukey post hoc test

^‡^Observed difference is clinically relevant, because difference exceeds 0.5 standard deviation

**Fig. 2:**
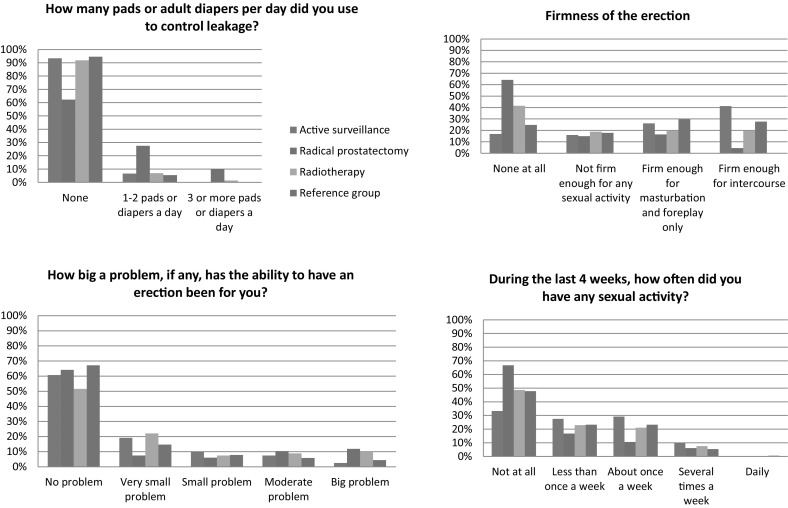
EPIC items on urinary and sexual functioning of relevance for use in daily clinical practice

The AS (*M* = 90.0, SD = 14.6) and reference (*M* = 90.4, SD = 13.9) groups less often reported urinary incontinence than the RP (M = 70.1, SD = 28.8) and RT groups (*M* = 86.5, SD = 20.3) [*F* (3,608) = 17.2, *p* < 0.001]. Comparing RT to RP, the RT group reported less urinary incontinence (*M* = 86.5, SD = 20.3 vs. *M* = 70.1, SD = 28.8). Differences between the AS&RP (*p* < 0.001) and RP&RT (*p* < 0.001) groups were statistically significant. In the RP group, 38% reported to use pads or diapers on a daily basis, versus 8% in the RT group, 7% in the AS group, and 5% in the reference group (Fig. 2). The urinary bother scores of the four groups were rather similar.

#### Bowel domain

Mean bowel function scores of men with PCa (AS: *M* = 95.7, SD = 6.0; RP: *M* = 92.6, SD = 9.2; RT: *M* = 93.0, SD = 9.1) were similar to that of men without PCa (*M* = 94.4, SD = 8.5) (Table 2).

#### Sexuality domain

Significant and clinically relevant differences were seen in the overall comparison (Sexual summary: *F* (3,605) = 4, *p* = 0.01; Sexual function: *F* (3,555) = 6.8, *p* < 0.001) as well as in AS&RP (sexual summary *p* = 0.01, sexual function *p* < 0.001) and RP&RT (sexual function *p* = 0.05) comparisons. The sexual function score was highest in men on AS (*M* = 40.9. SD = 24.6) and lowest for men who underwent RP (M = 14.8, SD = 17.7), indicating worst function. Sexual bother scores did not differ statistically between groups (Table 2), but large differences were seen with respect to the firmness of the erection and the amount of any sexual activity (Fig. 2).

### Generic health

The SF-12 PCS score differed significantly between the four groups (*F* (3,558) = 3.2, *p* = 0.02) (Table 2). The ANOVA Tukey post hoc test showed that this difference was caused by the difference between the AS vs. RT comparison (*M* = 50.7, SD = 6.6 vs. *M* = 47.8, SD = 8.3, *p* = 0.01) with the AS group reporting better physical functioning. Observed differences were not clinically relevant. No significant difference was seen for the mental summary score. On the EQ-VAS, men on AS rated their health best (AS: *M* = 81.8, SD = 12.7; RP *M* = 78.6, SD = 15.0; RT: *M* = 78.3, SD = 14.1; Reference: *M* = 79.5, SD = 12.4) (Table 2).

### Generic anxiety

Reported levels of generic anxiety were comparable amongst the four groups. The percentage of men that may be regarded as highly anxious (STAI score ≥ 44) amounted to 8% (10/120) in the AS group, 13% (9/69) in the RP group, 12% (27/219) in the RT group, and 12% (24/204) in the reference group (Table 2).

### Comparing active treatment after adjusting for clinical parameters

After correcting for age, PSA at diagnosis, cTstage, Gleason score, and follow-up time statistically, significant differences were found with respect to urinary function, urinary incontinence, urinary irritative, and sexual function (Table 3). Bonferroni post hoc test revealed that for urinary function and incontinence, it was the AS&RP and RP&RT groups that significantly differed from each other. For the urinary irritative domain, the RP group had a better score than the RT group (*M* = 99.1, SD = 1.03 vs. *M* = 90.2, SD = 1.02, *p* = 0.02). Comparing sexual functioning, the AS group reported the best score (*M* = 28.2, SD = 1.14), and the RP group the worst (*M* = 12.2, SD = 1.22) (AS&RP *p* = 0.002, RP&RT *p* = 0.05).

**Table 3:** Quality of life in the active surveillance (AS), radical prostatectomy (RP), and radiotherapy groups (RT) corrected for clinical characteristics (age, PSA at diagnosis, Gleason, cTstage, and follow-up)

	AS Adjusted mean and SD	RP Adjusted mean and SD	RT Adjusted mean and SD	All groups *P* value*	AS vs. RP *P* value^#^	AS vs. RT *P* value^#^	RP vs. RT *P* value^#^
Generic health							
SF12-PCS	49.6 (1.02)	48.3 (1.03)	47.2 (1.01)	0.126	1.00	0.129	1.00
SF12-MCS	52.8 (1.02)	51.4 (1.03)	54.2 (1.02)	0.225	1.00	0.993	0.294
Respondent’s self-reported health							
EQ-VAS	79.8 (1.02)	78.3 (1.03)	76.7 (1.02)	0.334	1.00	0.423	1.00
Generic anxiety							
STAI-6	30.7 (1.03)	31.4 (1.04)	32.4 (1.02)	0.283	1.00	0.348	1.00
Prostate-specific health							
EPIC							
Urinary summary	93.3 (1.02)	84.7 (1.03)	87.9 (1.02)	*0.016*	*0.022*	0.079	0.869
Urinary function	91.8 (1.02)	77.8 (1.03)	88.1 (1.02)	<*0.001*	<*0.001*	0.436	*0.001*
Urinary bother	92.5 (1.03)	92.3 (1.04)	89.7 (1.02)	0.682	1.00	1.00	1.00
Urinary incontinence	88.3 (1.05)	61.4 (1.06)	83.9 (1.04)	<*0.001*	<*0.001*	1.00	<*0.001*
Urinary irritative	95.5 (1.02)	99.1 (1.03)	90.2 (1.02)	*0.012*	0.956	0.138	*0.020*
Bowel summary	99.1 (1.03)	96.8 (1.04)	96.6 (1.02)	0.702	1.00	1.00	1.00
Bowel function	98.2 (1.03)	94.6 (1.04)	94.8 (1.02)	0.543	1.00	0.902	1.00
Bowel bother	97.1 (1.03)	98.6 (1.04)	100.0 (1.02)	0.635	1.00	1.00	1.00
Sexual summary	48.8 (1.07)	33.5 (1.10)	41.6 (1.06)	*0.010*	*0.007*	0.268	0.180
Sexual function	28.2 (1.14)	12.2 (1.22)	21.3 (1.11)	*0.003*	*0.002*	0.329	*0.045*
Sexual bother	80.9 (1.06)	77.4 (1.08)	78.3 (1.05)	0.893	1.00	1.00	1.00

*SD* standard deviation

*Significance tested with ANCOVA with covariates age, PSA at diagnosis, Gleason, cTstage, and follow-up

^#^ANCOVA Bonferroni post hoc test

## Discussion

In this paper, long-term patient reported QoL of men on AS, men who underwent RP or RT, and a reference group of men without prostate cancer was compared. Generic anxiety levels were similarly low for all four groups. In this observational study, for the first time, condition-specific function was measured with a single patient reported outcome measure (EPIC-26) across three treatment groups and a reference group without PCa. Statistically significant differences were found regarding urinary function and urinary incontinence with the AS group reporting better urinary function and less incontinence compared to the RP group. A statistically and clinically relevant (≥0.5SD) difference was found regarding sexual function. When comparing AS to RT, a statistically significant difference was seen regarding sexual function, with the AS group reporting better sexual function. This difference was, however, not clinically relevant. These observed differences in prostate-specific functioning between treatment groups could be expected, as AS has a less invasive character than RP and RT. Results furthermore indicate that the QoL of men on AS was very comparable to that of men without PCa.

Our data showed that only few men, i.e., 8% in the AS group, 12% in the RT and reference group, and 13% in the RP group, reported high levels of anxiety. There is a tendency to assume that men who choose AS and therewith live with untreated cancer, combined with the potential of missing the window of curability, will experience anxiety. Observational studies have shown results of AS cohorts in which anxiety levels are low [22, 23] versus studies in which anxiety levels of around 20% were seen [9, 24]. These observational studies involved selected groups of patients who chose to follow an AS strategy. In the recently published ProtecT trial, a randomized controlled trial in which men were randomly allocated to either active monitoring, RP or RT, longitudinal 5 year follow-up results were presented. With respect to anxiety, measured through the Hospital Anxiety and Depression Scale (HADS), no significant differences among the treatment groups were seen (*p* = 0.27), suggesting that men on AS are not more anxious than men on RP or RT [25]. This might indicate that psychological support is less needed for men on AS. However, it should be noted that only 62% (1643/2664) of eligible men underwent randomization in the ProtecT trial, while 38% selected their own treatment (based on own or urologist preferences). Furthermore, men who were randomized to active monitoring had regular contact with a specialist nurse with whom any concerns could be discussed. Furthermore, our data confirm the results that Bellardita et al. describe in their review study [8], summarizing findings of mainly short-term studies.

Although the RP group reported frequent urinary incontinence and both RP and RT groups reported limited sexual function, they reported similar levels of bother as the AS and reference groups, with the latter reporting hardly any urinary incontinence and better sexual functioning. The pattern of impaired function but no related bother can possibly be explained by response shift [26, 27]. Sprangers and Schwartz describe response shift as “a change in the meaning of one’s self-evaluation of QoL as a result of a change in one’s internal standards of measurement (i.e., recalibration), the importance attributed to component domains constituting QoL (i.e., change in values), or construal of the meaning of QoL (i.e., concept redefinition) [26, 28].” It may be that over time men have accepted the changes in, e.g., their sexual functioning, because they perceived these as an inevitable consequence of PCa treatment; a condition thought to be life threatening by at least a part of men with PCa [27, 29]. As a result, men may have changed the importance they attach to sexual activity to soften the negative influence of deteriorating sexual function (i.e., change in values) [28]. Behavioural scientists tend to refer to the response shift process as cognitive adaptation [30, 31]. Ageing may play a role as well [32]. In addition to the higher prevalence of erectile dysfunction in older men, Korfage et al. found that diminishing sexual activity with increasing age was considered more or less normal amongst a group of treated PCa patients [27, 33].

While patients are often able to adapt to a new situation and accept side-effects, it is important to enable them to consider potential consequences of treatment beforehand. We want to provide relevant PCa treatment information, and therewith enable men to base their treatment-choice on what they consider important and to avoid those side-effects they find least acceptable. The here presented outcomes for the three treatments can support men and their physicians in deciding what treatment fits the patient best in terms of QoL. By discussing, amongst others, the side-effects of each type of treatment as well as patients’ preferences, we furthermore hope to stimulate shared decision-making between patients and physicians, as patient participation may positively affect treatment decision-making. A recent study amongst 1529 men with clinically localized PCa explored whether active patient involvement in decision-making and greater patient knowledge are associated with better treatment decision-making experiences and QoL [34]. The authors concluded that the men who were knowledgeable about PCa and the side-effects of treatment at the time of treatment decision-making experienced better QoL 6 months after treatment because of realistic expectations regarding side-effects [34].

This study has limitations. Differences related to baseline clinical and demographic characteristics of the participants were seen. Because men were not randomized into treatment groups, clinical and demographic characteristics slightly varied per group. However, the study design we report does represent current clinical practice, where choice for treatment is selective and individualized. Our findings furthermore highlight the importance and need of a baseline, i.e., pre-diagnostic biopsy, measurement. Future studies, such as the mixed-method study by Ruane-McAteer et al. [35], will, by including such a baseline measurement, provide further valuable information on the impact on QoL of either AS or direct curative treatment on both mental and physical health of the patient. The RT group in our study is heterogeneous. As we acknowledge that there is a difference between brachytherapy and external radiation, we stratified QoL outcomes in the individual radiotherapy groups (i.e., mono brachytherapy and brachytherapy followed by external radiation and Cyberknife) in appendix Table 4. When comparing mono brachytherapy with brachytherapy + external radiation, mainly, a difference on the sexuality domain was seen with the mono brachytherapy group showing better sexual function. In comparing the brachytherapy + external radiation and Cyberknife groups, significantly higher bowel domain scores were seen for the Cyberknife group indicating better bowel function in the latter group. We furthermore chose an age-matched cohort of screening arm participants without PCa from the ERSPC Rotterdam study to reflect the general population. The previous studies have shown that ERSPC-Rotterdam participants differ from the general population, in the sense that they seem to be healthier [36, 37]. However, the entire RP group, 20% of the AS group, and 37% of the RT group also participated in ERSPC Rotterdam. Strengths of our study are the considerable length of follow-up of 4–10 years, the good questionnaire response rates, the use of one validated patient reported outcome measure—the EPIC—to compare prostate-specific function across all four groups, the inclusion of a non-PCa reference group, and, for the first time, the reporting of long-term QoL results for an AS cohort.

To conclude, in this cross-sectional study, we compared long-term QoL of PCa patients on AS to that of PCa patients who followed direct curative regimens and a reference group of men without PCa. Generic anxiety was similarly low amongst all four groups and in terms of prostate-specific functioning, the AS group reported better sexual function as compared to the RP and RT groups and better urinary function than the RP group. The QoL of men on AS was very comparable to that of men without PCa. Our results indicate men who followed an AS strategy for a long-term period were not anxious and accepted it well, suggesting that AS may be a good treatment option for men with low-risk PCa.

## Appendix

See Table 4.

**Table 4:** Questionnaire scores per RT-type

	Score range	HDR-brachy + external *N* = 121	HDR-brachy *N* = 73	Cyberknife *N* = 38		Brachy + external vs. brachy	Brachy + external vs. cyberknife	Brachy vs. cyberknife
		Mean (SD)	Mean (SD)	Mean (SD)	*P* value*	*P* value^#^	*P* value^#^	*P* value^#^
Generic health								
SF-12—PCS	0–100	46.6 (8.3)	48.7 (7.6)	49.7 (9.3)	0.191	0.111	0.186	0.824
SF-12—MCS	0–100	53.6 (8.9)	54.3 (8.6)	58.3 (6.0)	*0.028*	0.609	*0.008*	*0.019*
EQ-VAS	0–100	77.4 (13.9)	79.0 (14.8)	79.4 (13.5)	0.772	0.571	0.549	0.873
STAI-6	20–80	34.1 (9.5)	32.9 (7.9)	31.0 (6.2)	0.273	0.493	0.121	0.272
Highly anxious (N, %)	≥44	18 (14.9%)	8 (11.0%)	1 (2.6%)				
EPIC								
Urination	0–100, with higher scores indicating better quality of life							
Urinary summary		88.8 (16.3)	89.8 (13.2)	91.7 (12.1)	0.496	0.513	0.289	0.489
Urinary function		88.4 (18.4)	90.3 (14.3)	92.6 (11.4)	0.294	0.352	0.178	0.376
Urinary bother		89.0 (16.2)	89.4 (13.9)	91.1 (13.9)	0.724	0.702	0.459	0.598
Urinary incontinence		84.9 (22.3)	86.6 (19.1)	91.5 (14.7)	0.223	0.453	0.105	0.195
Urinary irritative		91.8 (13.9)	92.9 (10.5)	91.9 (12.3)	0.708	0.416	0.837	0.621
Bowel								
Bowel summary		93.3 (8.8)	95.4 (7.4)	97.5 (3.0)	*0.024*	0.133	*0.010*	0.136
Bowel function		91.3 (10.6)	94.0 (7.7)	96.0 (4.5)	*0.020*	0.073	*0.019*	0.181
Bowel bother		95.3 (8.5)	96.7 (7.8)	99.0 (2.9)	0.075	0.341	*0.019*	0.135
Sexuality								
Sexual summary		37.8 (19.6)	45.0 (20.2)	43.2 (21.1)	0.068	*0.018*	0.242	0.645
Sexual function		21.0 (23.9)	31.3 (26.0)	29.2 (23.9)	0.069	*0.058*	0.069	0.752
Sexual bother		75.7 (26.6)	76.6 (24.8)	74.4 (31.3)	0.992	0.922	0.913	0.969

*SD* standard deviation

*3-group comparison: significance tested with ANOVA after log transformation of the scores

^#^2-group comparisons: significance tested with T test after log transformation of the scores

## References

[1] Sanda MG, Dunn RL, Michalski J, Sandler HM, Northouse L, Hembroff L, Lin X, Greenfield TK, Litwin MS, Saigal CS, Mahadevan A, Klein E, Kibel A, Pisters LL, Kuban D, Kaplan I, Wood D, Ciezki J, Shah N, Wei JT (2008). Quality of life and satisfaction with outcome among prostate-cancer survivors. The New England Journal of Medicine.

[2] Cooperberg MR, Carroll PR (2015). Trends in management for patients with localized prostate cancer, 1990–2013. JAMA: The Journal of the American Medical Association.

[3] Tran K, Rahal R, Fung S, Louzado C, Porter G, Xu J, Bryant H, Collaboration with the System Performance Steering, C., Technical Working, G (2016). Patterns of care and treatment trends for Canadian men with localized low-risk prostate cancer: an analysis of provincial cancer registry data. Curr Oncol.

[4] Klotz L, Vesprini D, Sethukavalan P, Jethava V, Zhang L, Jain S, Yamamoto T, Mamedov A, Loblaw A (2015). Long-term follow-up of a large active surveillance cohort of patients with prostate cancer. Journal of Clinical Oncology: Official Journal of the American Society of Clinical Oncology.

[5] Heidenreich A, Bastian PJ, Bellmunt J, Bolla M, Joniau S, van der Kwast T, Mason M, Matveev V, Wiegel T, Zattoni F, Mottet N (2014). EAU guidelines on prostate cancer. part 1: screening, diagnosis, and local treatment with curative intent-update 2013. European urology.

[6] Thompson I, Thrasher JB, Aus G, Burnett AL, Canby-Hagino ED, Cookson MS, D’Amico AV, Dmochowski RR, Eton DT, Forman JD, Goldenberg SL, Hernandez J, Higano CS, Kraus SR, Moul JW, Tangen CM, Panel AUAPCCGU (2007). Guideline for the management of clinically localized prostate cancer: 2007 update. The Journal of Urology.

[7] Bruinsma SM, Bangma CH, Carroll PR, Leapman MS, Rannikko A, Petrides N, Weerakoon M, Bokhorst LP, Roobol MJ, Movember GAPc (2016). Active surveillance for prostate cancer: a narrative review of clinical guidelines. Nature Reviews Urology.

[8] Bellardita L, Valdagni R, van den Bergh R, Randsdorp H, Repetto C, Venderbos LD, Lane JA, Korfage IJ (2015). How does active surveillance for prostate cancer affect quality of life? A systematic review. European urology.

[9] Watts S, Leydon G, Eyles C, Moore CM, Richardson A, Birch B, Prescott P, Powell C, Lewith G (2015). A quantitative analysis of the prevalence of clinical depression and anxiety in patients with prostate cancer undergoing active surveillance. BMJ Open.

[10] Venderbos, L. D., van den Bergh, R. C., Roobol, M. J., Schroder, F. H., Essink-Bot, M. L., Bangma, C. H., Steyerberg, E. W., & Korfage, I. J. (2014). A longitudinal study on the impact of active surveillance for prostate cancer on anxiety and distress levels. *Psychooncology*. 10.1002/pon.3657 25138075

[11] Marteau TM, Dormandy E, Michie S (2001). A measure of informed choice. Health Expectations: An International Journal of Public Participation in Health Care and Health Policy.

[12] van den Bergh RC, Roemeling S, Roobol MJ, Roobol W, Schroder FH, Bangma CH (2007). Prospective validation of active surveillance in prostate cancer: the PRIAS study. European urology.

[13] Bokhorst, L. P., Alberts, A. R., Rannikko, A., Valdagni, R., Pickles, T., Kakehi, Y., Bangma, C. H., Roobol, M. J., & group, P. s. (2015). Compliance rates with the prostate cancer research international active surveillance (PRIAS) protocol and disease reclassification in noncompliers. *Eur Urol*. 10.1016/j.eururo.2015.06.012 26138043

[14] Schroder FH, Denis LJ, Roobol M, Nelen V, Auvinen A, Tammela T, Villers A, Rebillard X, Ciatto S, Zappa M, Berenguer A, Paez A, Hugosson J, Lodding P, Recker F, Kwiatkowski M, Kirkels WJ, Erspc (2003). The story of the European Randomized Study of Screening for Prostate Cancer. BJU International.

[15] Wei JT, Dunn RL, Litwin MS, Sandler HM, Sanda MG (2000). Development and validation of the expanded prostate cancer index composite (EPIC) for comprehensive assessment of health-related quality of life in men with prostate cancer. Urology.

[16] van Tol-Geerdink JJ, Leer JW, van Oort IM, van Lin EJ, Weijerman PC, Vergunst H, Witjes JA, Stalmeier PF (2013). Quality of life after prostate cancer treatments in patients comparable at baseline. British Journal of Cancer.

[17] Ware J, Kosinski Jr, &amp, Keller, S D (1996). A 12-Item Short-Form Health Survey: construction of scales and preliminary tests of reliability and validity. Medical care.

[18] van Reenen M, Janssen B, for the EuroQoL group. ‘EQ-5D-5L user guide. Basic information on how to use the EQ-5D-5L instrument’. April 2015, version 2.1. Accessed through http://www.euroqol.org/fileadmin/user_upload/Documenten/PDF/Folders_Flyers/EQ-5D-5L_UserGuide_2015.pdf.

[19] Marteau TM, Bekker H (1992). The development of a six-item short-form of the state scale of the Spielberger State-Trait Anxiety Inventory (STAI). The British Journal of Clinical Psychology/The British Psychological Society.

[20] Millar K, Jelicic M, Bonke B, Asbury AJ (1995). Assessment of preoperative anxiety: comparison of measures in patients awaiting surgery for breast cancer. British Journal of Anaesthesia.

[21] Norman GR, Sloan JA, Wyrwich KW (2003). Interpretation of changes in health-related quality of life: the remarkable universality of half a standard deviation. Medical care.

[22] Venderbos LD, van den Bergh RC, Roobol MJ, Schroder FH, Essink-Bot ML, Bangma CH, Steyerberg EW, Korfage IJ (2015). A longitudinal study on the impact of active surveillance for prostate cancer on anxiety and distress levels. Psycho-oncology.

[23] Vasarainen H, Lokman U, Ruutu M, Taari K, Rannikko A (2012). Prostate cancer active surveillance and health-related quality of life: results of the Finnish arm of the prospective trial. BJU International.

[24] Simpkin AJ, Tilling K, Martin RM, Lane JA, Hamdy FC, Holmberg L, Neal DE, Metcalfe C, Donovan JL (2015). Systematic review and meta-analysis of factors determining change to radical treatment in active surveillance for localized prostate cancer. European urology.

[25] Donovan, J. L., Hamdy, F. C., Lane, J. A., Mason, M., Metcalfe, C., Walsh, E., Blazeby, J. M., Peters, T. J., Holding, P., Bonnington, S., Lennon, T., Bradshaw, L., Cooper, D., Herbert, P., Howson, J., Jones, A., Lyons, N., Salter, E., Thompson, P., Tidball, S., Blaikie, J., Gray, C., Bollina, P., Catto, J., Doble, A., Doherty, A., Gillatt, D., Kockelbergh, R., Kynaston, H., Paul, A., Powell, P., Prescott, S., Rosario, D. J., Rowe, E., Davis, M., Turner, E. L., Martin, R. M., Neal, D. E., & Protec, T. S. G. (2016). Patient-reported outcomes after monitoring, surgery, or radiotherapy for prostate cancer. *N Engl J Med*. 10.1056/NEJMoa1606221 PMC5134995 27626365

[26] Sprangers MA, Schwartz CE (1999). Integrating response shift into health-related quality of life research: a theoretical model. Social Science and Medicine (1982).

[27] Korfage IJ, Hak T, de Koning HJ, Essink-Bot ML (2006). Patients’ perceptions of the side-effects of prostate cancer treatment–a qualitative interview study. Social Science and Medicine (1982).

[28] Lepore S.J., & Eton D.T. (2000). Response shifts in prostate cancer patients: an evaluation of suppression and buffer models. In C. E. Schwartz and M. A. G. Sprangers (Eds.), *Adaptation to changing health—response shift in quality-of-life research* (pp.37–51). American Psychological Association; 2000. ISBN: 978-1-55798-710-5.

[29] Korfage IJ, de Koning HJ, Essink-Bot ML (2007). Response shift due to diagnosis and primary treatment of localized prostate cancer: a then-test and a vignette study. Quality of Life Research: An International Journal of Quality of Life Aspects of Treatment, Care and Rehabilitation.

[30] Taylor SE (1983). Adjustment to threatening events—a theory of cognitive adaptation. American Psychologist.

[31] Sinclair VG, Blackburn DS (2008). Adaptive coping with rheumatoid arthritis: the transforming nature of response shift. Chronic Illness.

[32] Nicolosi A, Laumann EO, Glasser DB, Behaviors, Investigators, G (2004). Sexual behavior and sexual dysfunctions after age 40: the global study of sexual attitudes and behaviors. Urology.

[33] Korfage IJ, Roobol M, de Koning HJ, Kirkels WJ, Schroder FH, Essink-Bot ML (2008). Does “normal” aging imply urinary, bowel, and erectile dysfunction? A general population survey. Urology.

[34] Orom, H., Biddle, C., Underwood, W. 3rd, Nelson, C. J., & Homish, D. L. (2016). What is a “good” treatment decision? Decisional control, knowledge, treatment decision making, and quality of life in men with clinically localized prostate cancer. Medical Decision Making. 10.1177/0272989X16635633 PMC4930707 26957566

[35] Ruane-McAteer E, O’Sullivan J, Porter S, Venderbos L, Prue G (2016). An exploration of men’s experiences of undergoing active surveillance for favourable-risk prostate cancer: A mixed methods study protocol. BMC Cancer.

[36] Otto SJ, Schroder FH, de Koning HJ (2004). Low all-cause mortality in the volunteer-based Rotterdam section of the European randomised study of screening for prostate cancer: self-selection bias?. Journal of Medical Screening.

[37] Nijs HG, Tordoir DM, Schuurman JH, Kirkels WJ, Schroder FH (1997). Randomised trial of prostate cancer screening in The Netherlands: assessment of acceptance and motives for attendance. Journal of Medical Screening.

